# Molecular and Circulating Biomarkers of Gastric Cancer

**DOI:** 10.3390/ijms23147588

**Published:** 2022-07-08

**Authors:** Wojciech Jelski, Barbara Mroczko

**Affiliations:** 1 Department of Biochemical Diagnostics, Medical University, 15-269 Bialystok, Poland; barbara.mroczko@umb.edu.pl; 2 Department of Neurodegeneration Diagnostics, Medical University, 15-269 Bialystok, Poland

**Keywords:** tumor biomarkers, gastric cancer

## Abstract

Gastric cancer (GC)—a common tumor that affects humans worldwide—is highly malignant with a poor prognosis. GC is frequently not diagnosed until a relatively advanced stage. Early detection and efficient monitoring of tumor dynamics are prerequisites for reducing disease burden and mortality. Minimally invasive methods are needed to establish a diagnosis or monitoring the response to treatment of gastric cancer. Blood-based biomarker assays for the detection of early-stage GC could be of great relevance both for the risk group or for population-wide based screening programs, The currently used tumor marker assays for detecting GC are simple and rapid, but their use is limited by their low sensitivity and specificity. In recent years, several markers have been identified and tested for their clinical relevance in the management of gastric cancer. Here we review the available literature on plasma classical tumor markers, circulating free microRNAs (cfmiRNAs), circulating cell-free DNA (cfDNA), circulating tumor cells (CTCs), autoantibodies against tumor associated antigens (TAAs), and circulating extracellular vesicles (EVs) for diagnosis and monitoring of gastric cancer. This review summarizes the present status and approaches for these biomarkers, which could be potentially used for early diagnosis and accurate prediction of therapeutic approaches. We also discuss the future perspective and challenges in the search for new biomarkers of gastric cancer.

## 1. Introduction

**Figure 1:**
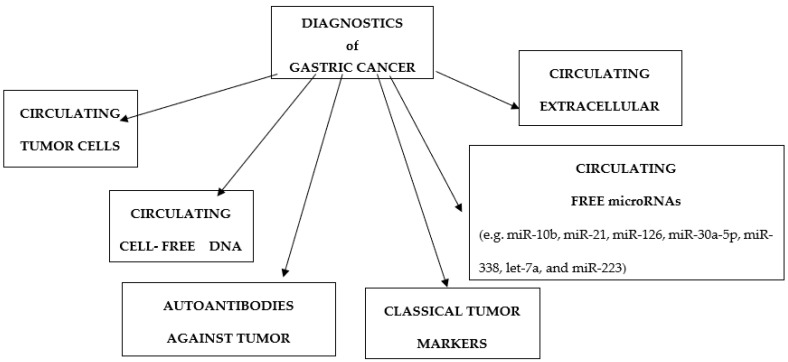
Division of gastric cancer markers.

Gastric cancer (GC) is one of the leading causes of cancer morbidity and mortality in the world. Each year, there are still over a million newly diagnosed cases and deaths due to stomach cancer despite the decline in incidence. The incidence rates vary significantly across the globe, being the highest in Eastern Asia, followed by Eastern and Central Europe while rates are the lowest in North America and Western Africa [1]. Although the main type of GC is adenocarcinoma (approximately 95%), gastric cancer is genetically and biologically heterogeneous with a poorly understood carcinogenesis at the molecular level. A relatively small percentage of GC cases is associated with nutritional factors and genetic predisposition, and the main risk factors are the presence of precancerous lesions (dysplasia) and chronic H. pylori infection [2,3]. The survival rate depends on the stage of gastric cancer at the time of the diagnosis. In Japan survival rate was found to be about 50%, whereas in Western countries the 5-year survival ranged from 5–20% due to late diagnosis [4]. As patients with early GC typically have no symptoms, they often miss the opportunity for optimal treatment. Although surgical resection, along with chemotherapy and radiotherapy, is more effective than surgery alone in patients with early-stage gastric cancer, patients often present with late-stage cancer at initial diagnosis due to the absence of clinical symptoms which could have enabled early detection [5]. Gastroscopy with biopsy is an appropriate method assisting in the diagnosis of specific early gastric tumor types; but the stress caused by this invasive method together with the fact that it is very expensive, make it difficult to use it as a routine method of screening for gastric cancer [6]. A prerequisite for reducing mortality and improving treatment in gastric cancer patients is early detection and monitoring of tumor dynamics [2]. So, it is still a huge challenge to detect gastric cancer at the early stages because of the vacancy of specific detection tests. Hence, there is a great need to discovery biomarkers for the non-invasive early detection of gastric cancer patients. As detection of blood tumor markers are more convenient than other approaches, they are widely applied in the early diagnosis of gastric cancer. There have been lots of investigations to find the accurate serum and tumor biomarkers to detect gastric cancer. However, the sensitivity and specificity of currently used serum biomarkers for GC detection are unfavorable. In addition, relevant markers for monitoring prognosis have not yet been identified. Therefore, the first imperative is to investigate new effective GC biomarkers to aid in early diagnosis and guide treatment planning [2]. Biomarkers are characteristics that are objectively evaluated and measured as an indicator of normal or pathological biologic processes or pharmacological response to therapy [7]. Commonly used tumor markers are carcino-embryonic antigen (CEA), carbohydrate antigen (CA 19-9), and carbohydrate antigen 72-4 (CA72-4). However, none of them demonstrates a high level of diagnostic accuracy [8]. Recent research to identify GC biomarkers has resulted in the discovery of a wide variety of cancer-related molecules, including various proteins, circulating free microRNAs (cfmiRNAs), circulating cell-free DNA (cfDNA), circulating tumor cells (CTCs), autoantibodies against tumor associated antigens (TAAs), and cancer-derived extracellular vesicles (EVs) (Figure 1, Table 1) [9].

## 2. Classical Tumor Markers

A “liquid biopsy” for GC patients is used to detect physiological indicators in the blood. This procedure is less invasive than endoscopic or surgical biopsy, allows for earlier disease detection and monitoring of gastric tumor development, as well as resistance to chemotherapy and treatment effectiveness. Serum tumor markers are widely applied in the diagnosis, treatment effect assessment, and disease monitoring. Although many gastric cancer biomarkers including carbohydrate antigen 72-4 (CA 72-4), alpha-fetoprotein (AFP), carbohydrate antigen 125 (CA 125), β-subunit of human chorionic gonadotropin (β-hCG), and pepsinogen I/II have been described, carcinoembryonic antigen (CEA) and carbohydrate antigen 19-9 (CA19-9) are still the most frequently used biomarkers in clinical practice for gastric cancer.

### 2.1. Carcinoembryonic Antigen

CEA is a glycoprotein oncofetal antigen that is expressed in many epithelial tumors. It is a cell-surface-anchored protein involved in cell–cell adhesions and it is a functional receptor for the ligands E-selectin of colorectal cancer and L-selectin, which may be critical in the metastatic spread of colon cancer cells [10]. In clinical practice in gastrointestinal cancer, CEA is the most commonly used marker. CEA is primarily used as a biomarker to monitor colorectal cancer treatment and to detect recurrences after surgical resection. CEA concentration may also increase in other types of cancer and in some non-cancerous conditions. High levels of carcinoembryonic antigen are found in high-stage GC in a large proportion of all patients; therefore, the level of this marker is not an effective screening method. Measurement of CEA mRNA by reverse transcription polymerase chain reaction (RT-PCR) can be used to detect micrometastases in the peritoneal cavity [11].

### 2.2. Carbohydrate Antigen 19-9

CA19-9 is a widely used marker for colorectal cancer; however, it occurs in many types of cancer, particularly gastric and pancreatic cancer. CA19-9-positive GCs showed distinct clinico-pathological features such as antral localization, advanced grade, differentiated histology, and a higher percentage of lymph node metastases. One of the studies demonstrated that the specificity for recurrence of CA19-9 was 74%, with a sensitivity of 56% [12]. It was found that the sensitivity increased to 87% when CA19-9 was combined with CEA [13].

### 2.3. Carbohydrate Antigen 72-4

Carbohydrate antigen 72-4 is a mucin-like glycoprotein present on the surface of various tumor cells. CA72.4-Ab assay shows good specificity for gastric cancer, and it is used for follow-up after treatment and to identify GC relapses. Although CA72-4 often exhibits higher sensitivity and accuracy compared to CEA, there have not been many studies on the early detection of CA72-4 or predictive screening [14].

### 2.4. Others Classical Tumor Markers

Biomarkers of tumor, such as AFP, β-subunit of human chorionic gonadotropin (β-hCG), CA125, and cytokeratin subunit 19 fragment (Cyfra21.1) have been widely used for the diagnosis of gastric cancer. However prognostic significance of these markers for early gastric cancer has not yet been investigated. These tumor markers are not optimal for gastric cancer screening due to their low sensitivity and specificity [15].

Alpha-fetoprotein producing gastric carcinoma (AFPGC) is a rare type of gastric cancer with high malignancy and poor prognosis, which makes it different from other types of gastric cancer. AFPGC refers to the serum and gastric cancer tissue containing a large amount of AFP with the exclusion of other possible diseases (hepatocellular carcinoma, active liver disease, genitourinary system tumors) that may produce AFP. Therefore, it is recommended that physicians routinely examine the level of serum AFP in gastric cancer patients, especially in patients with liver metastasis, while pathological specimens of routine AFP immunohistochemistry can greatly improve the detection rate of GC [16].

Pretreatment serum CA125 is a useful prognostic biomarker in patients with unresectable advanced or recurrent gastric cancer. CA125 level has been said to be significantly associated with the occurrence of peritoneal dissemination in GC [3]. In patients who have carried out curative surgery, CA125 positivity may serve as the predictor of peritoneal dissemination. CA125 might be an important biomarker for evaluating patient outcomes and predicting prognosis more precisely, not only for patients who have undergone curative surgery for gastric cancer, but also for patients with unresectable advanced or recurrent gastric cancer who have been treated with systemic chemotherapy, particularly if it is used with other tumor markers [17].

The specificity and sensitivity of currently applicable blood biomarkers for the detection of GCs such as carcinoembryonic antigen, carbohydrate antigen 19-9, and carbohydrate antigen 72-4 are unfavorable. Higher levels of sensitivity and specificity can be obtained when testing several markers together. A combination of serum CEA and CA19–9 has been indicated to obtain higher specificity than serum CEA alone. Moreover, the combination of CEA, CA125, and CA19–9 has been reported to attain higher sensitivity than CEA alone [18].

## 3. Circulating Tumor Cells

Circulating tumor cells (CTCs) are cells released from the primary tumor into the vascular system that circulate in the bloodstream. Cells can be released from the original tumor and/or corresponding distant metastatic sites. Generally, CTCs released into the circulation have a short lifetime and only a few highly active tumor cells with high metastatic potential survive in the circulation. Circulating tumor cell capture provides real-time access to neoplastic tissues without the need for invasive biopsy, and their phenotypic and molecular examination can provide insight into the biological changes in the tumor occurring during treatment [19]. CTCs have proved to be a reliable source of neoplastic cells, and their concentration has turned out to be of prognostic importance. The CTC has already been approved by the Food and Drug Administration (FDA) as a prognostic biomarker for monitoring patients with breast, prostate, and colon cancer. Circulating tumor cells may also play a key role in monitoring the spread of GC and treating patients with relapsed and metastatic gastric cancer [20].

The CTCs can be collected and detected using appropriate technologies according to their biological and physical properties. The CTC detection process mainly includes separation, enrichment and identification. After the enrichment stage, we can increase significantly CTC concentration and allow easy detection of even a single neoplastic cell. Various techniques can be used to detect CTCs. Tumors of epithelial origin express the epithelial cell adhesion molecule (EpCAM) and cytokeratin (CK) antigen. The CELLSEARCH^®^ Circulating Tumor Cell Kit, a product of Johnson (Veridex), is currently the world’s first and only FDA approved CTC detection kit intended for the enumeration of CTCs of epithelial origin (EpCAM+, CD45-, and cytokeratins 8, 18+, and/or 19+) in whole blood. Some studies have reported the clinical value of CTCs as prognostic markers by other detection methods, including the RT-PCR, but the number of studies using this method is relatively small [21]. Uenosono et al. detected CTCs using the CellSearch system in 251 patients with GC and showed that overall survival (OS) was significantly lower in people with CTCs than in patients without CTC (*p* < 0.001) [22]. In another study, Matsusaka et al. found a correlation between CTCs and clinical outcomes and chemotherapy outcomes. They showed that patients with GC with at least four CTCs at 2 and 4 weeks after the initiation of chemotherapy had significantly shorter overall and progression-free survival than patients with less than four CTCs. The chemotherapy regimen was an S-1-based regimen (S-1 with or without cisplatin) or paclitaxel [23]. In addition, Liu et al. showed that after the first cycle of chemotherapy, patients with an increase in the number of CTCs developed tumor progression and that patients with a decreasing number of CTCs achieved a complete, partial or stable disease response [24].These results may indicate that the response to treatment with CTCs is correlated with clinical outcomes.

Circulating tumor cells from cancer patients can be thought of as a type of real-time “liquid biopsy” that can provide real-time information about the cancer status. In addition to the CellSearch system, new and more sensitive experimental approaches are being developed for the detection of rare CTCs. However, data on their sensitivity to early GC are still limited. The scarcity of knowledge has hampered the progress of the use of circulating tumor cells in clinical diagnostics. However, significant new perspectives have emerged regarding the biological importance of CTCs and various revolutionary techniques [25].

## 4. Circulating Free miRNA

The miRNA consists of 20–24 nucleotides. They are a class of non-coding small molecule single chain RNAs and have highly temporal, conservative and tissue specific characteristics. They are common in eukaryotes and regulate cell differentiation, proliferation, and apoptosis [26]. The role of miRNA in the progression and development of cancer cells is based on differentiation, modulating growth, and apoptosis processes. One type of miRNA can regulate multi-target expression of genes and many pathways influencing the process of cancer development. Therefore, miRNAs are much more effective than gene encoding molecules as biological regulation molecules. Analysis of miRNA accurately identified tumor cell origin in a variety of tumors. Recently, due to the stability and specificity of expression in tissues and circulation, accumulating evidence has shown that miRN As can be regarded as novel biomarkers with a potential clinical significance tool for GC patient outcomes. Numerous researchers analyzed the serum miRNA signature of the GC as prognostic and diagnostic indicators. MiRNAs can be released from neoplastic tissues into body fluids, not only into serum and plasma, but also into gastric juice, tears, urine, and amniotic fluid by secreting exosome particles. Many Chinese and other research teams around the world have discovered numerous types of miRNAs that play a predictive role in gastric cancer [27]. Li et al. showed that a few-miRNA signature (miR-10b, miR-21, miR-126, miR-30a-5p, miR-338, let-7a, and miR-223) is an independent predictor of overall survival and relapse-free survival [28]. In addition, numerous research teams have discovered many miRNAs that play a role as biomarkers in stomach cancer. For example, high expression of miRNA-150, miRNA-20b, miRNA-142-5p, miRNA-214, and miRNA-375 and low expression of miRNA-433, miRNA-451, let7g, and miRNA-125-5p are associated with short survival time [29,30]. Low levels of miRNA-126, miRNA-148, miRNA-146a, miRNA-218, miRNA-429, and miRNA-335 and high levels of miRNA-27a and miRNA-650 indicate lymph node metastasis [31,32,33]. Usually, distant metastases often lead to advanced cancer and shorter survival. Therefore, oncomiR-10b, miR-21, and miR-212 in gastric cancer patients have been shown to be associated with a high risk of metastasis and poor clinical outcomes, including tumor size, lymph node metastases, stage, and a five-year survival rate [34].

Current miRNA-mediated therapies focus on miRNA knockout and silencing of endogenous oncomiRs, including miRNA sponges and anti-miRNA oligonucleotides [35]. For example, Chun et al. in their study transfected AS-miR-221/222 with liposomes into GC cell line SGC7901 to inhibit GC cell growth and invasion [36]. Here, we introduce identified miRNAs that potentially represent biomarkers for GC (Table 2) [37].

Real-time RT-PCR and microarrays are analytical techniques commonly used for validation and screening. Ideally, miRNAs could be a much better therapeutic tool than monogenic therapy because of their ability to target multiple genes. Unfortunately, several problems have arisen in trials of clinical use. Identification of the downstream targets of miRNA is intricate. Moreover, studies based on the clinical application of miRNAs for gastric cancer still lack accurate and reliable data from large-scale multi-center studies. However new miRNAs have been discovered and research techniques are constantly updated.

## 5. Circulating Extracellular Vesicles

Extracellular vesicles (EVs) are small (about 40–100 nm diameter) vesicles surrounded by a lipid bilayer that are released from both cancerous and non-cancerous tissues into the extracellular space. They play a major role in intercellular connectivity between the cancer and its surrounding stromal cells, and even between the tumor and distant cells. EVs carry various cellular components such as lipids, proteins, and nucleic acids (DNA, mRNA, non-coding RNA). Sometimes EVs are referred to as “exosomes” [38]. EVs can be found in a variety of bioliquids, including serum, plasma, cerebrospinal fluid, urine, and saliva. Much evidence suggests that EVs secreted by tumor cells affect surrounding cells and even cells at distal sites, thereby allowing tumor growth [39,40]. For example, exosomal integrins of the tumor can determine organotrophic metastases, and EVs secreted from gastric cancer also supply the epidermal growth factor receptor (EGFR), which may induce liver metastasis [41]. Moreover, Wu et al. found that GC EVs activate macrophages to promote cancer progression This is done by activating the NF-κB pathway and can provides a potential therapeutic approach in GC by disrupting interactions between exosomes and macrophages in the tumor microenvironment [42]. Tumor cells release exosomes that contain cancer-specific indicators and can detect the characteristics of the primary tumor. It has been found that exosomes isolated from bioliquids of cancer patients contain functional molecules derived from the tumor, which may be a powerful non-invasive diagnostic and prognostic tool for cancer. Studies have demonstrated the diagnostic potential of EV-derived cancer to detect different types of cancer, including colon, ovarian, prostate cancer, and melanoma [43]. A few years ago, a summary of the role of extracellular carriers or exosomes in gastric cancer was presented. This has resulted in an increase in the study of exosomes in the field of GC. EVs play a relatively important role in the tumorigenesis (metastasis, angiogenesis, immune escape) of GC. The mechanism of this action is mainly related to the specific load they carry. There is likely a bidirectional transfer of molecules between GC cells and the stromal cells in the tumor microenvironment, which helps to establish a niche against metastasis and to develop resistance to treatment [44]. The noninvasive nature, possibility for real-time assessment, and stable characteristics make EVs an ideal potential biomarker. In recent years, some exosomal proteins and miRNAs were found to be elevated in the blood of GC patients, showing that these EVs can be diagnostic markers for gastric cancer. As a result of a comparison of RNA sequencing analysis of plasma exosomes between five healthy subjects and 10 patients with stage I gastric cancer, lncUEGC1 and lncUEGC2 were confirmed to be significantly up-regulated in exosomes derived from patients with early-stage cancer. Plasma long noncoding RNA LINC00152 encompassed by exosomes is a stable potential indicator for gastric cancer [45]. Serum exosomal long noncoding RNA HOTTIP was significantly lower in 120 healthy controls than in 126 patients with GC p which suggested that HOTTIP is a novel potential diagnostic and prognostic biomarker test for gastric cancer [46]. In numerous studies, DNA or protein has been the focus of EV detection. Guo et al. conducted methylation detection using extracellular vesicles derived from gastric cancer cell lines, GC tissues, and gastric juice and found higher concentration of BarH-like 2 homeobox protein (BARHL2) methylation in gastric juice from patients with early stage of cancer and GC cell lines, with lower levels in gastritis (both normal and atrophis) [47].

Yoon et al. identified Gastrokine 1 (GKN1) through a protein microarray in 2018 and found that it binds to 27 EV proteins. GKN1 in EVs can inhibit the proliferation of a variety of GC cells and induce apoptosis. It was confirmed that GKN1 is a tumor suppressor that reduces GC initiation [48]. Wei et al. used qRT-PCR to show that miR-15b-3p is highly expressed in EVs, enhancing the tumorigenesis and malignant transformation of GC by inhibiting the NYDLT1/Caspase-3/Caspase-9 pathway and suppressing apoptosis in gastric cancer [49].

A recently published article claimed that EVs containing miR-6785-5p could suppress angiogenesis and metastasis in GC [50]. Studies have found that miR-130a, miR-135b, miR-155, miR-23a, X26nt, and YB-1 promote angiogenesis through different mechanisms. [51,52,53,54]. In addition, EVs containing secretory epidermal growth factor receptor (EGFR) derived from GC cells effectively activate hepatocyte growth factor, which in turn binds to c-MET receptors on migrating cancer cells to promote the homing of metastatic cancer cells [55]. Furthermore, M1 macrophage-derived EVs containing miR-16-5p were found to trigger a T cell immune response by decreasing the expression of PD-L1, which could eventually suppress tumor progression [56].

Most of the methods used to isolate exosomes today co-isolate heterogeneous populations of extracellular vesicles of diverse biogenic origins. The structure, related technologies, and mechanisms of exosomes are currently being explored and applied. Taking into consideration the current progress in this matter, further studies on EVs released in GC patients are needed and show promise to be successful.

## 6. Circulating Cell-Free DNA

Circulating cell-free DNA (cfDNA) is cell-free extracellular DNA originating from normal or cancerous cells identifiable in the serum. The fraction of cell-free DNA that derives from primary tumors or metastases and from CTCs is called ctDNA. cfDNA can be detected in the serum or plasma of healthy people, not only patients suffering from neoplastic diseases or other destructive diseases. Most of the circulating cell-free tumor DNAs is derived from apoptotic or necrotic tumor cells which are the source of fragmented DNA released into the circulating blood. Dying benign host tissues can also release cell-free DNAs into the blood. This normal circulating cell-free DNA can dilute the ctDNA levels in GC patients, especially when tissue-damaging procedures have been performed, including surgery, chemotherapy, or radiation therapy [57]. Numerous studies have shown that the level of ctDNA in the circulation of cancer patients is usually higher than in healthy subjects and cfDNA showed the same biological characteristics as the tissue tumor, suggesting that the cfDNA in tumor patients is mainly derived from ctDNA, whereas in healthy people, cfDNA is mainly derived from blood cells [58]. Currently, the most widely studied issue in cfDNA research is the utility of ctDNA in the treatment of cancer. Conventional biopsy causes significant trauma and allows for the collection of a small amount of sample. In contrast, detecting ctDNA has several benefits, including minimal invasiveness, convenient retrieval, and high repeatability [7]. Since measuring the level of circulating cell-free DNA does not require any a priori knowledge of the genetic changes in the tumor tissue, such an approach could be of great importance to develop non-invasive tests for the early detection of gastric cancer [59]. Some clinical applications of ctDNA have been exploited in gastric cancer. ctDNA is not only a tool for cancer detection in the early stage, but also a predictive or prognostic factor [60,61].

Park et al. showed that in their 54 GC patients and 59 age-matched healthy controls, the mean concentrations of plasma cfDNA were about 2.4-fold higher in the tested group compared with the control individuals, indicating that levels of cfDNA in the plasma may be useful for predicting patients with gastric cancer [60]. In another study Kim et al. and Sai et al. found that the cfDNA levels could distinguish between GC and the control group with an AUC varying from 0.750 to 0.991 respectively [61,62]. Kim et al. also showed that the level of cfDNA at 24 h after surgery significantly decreased compared to the preoperative values [61]. Unfortunately, elevated levels of cfDNA have also been detected in patients with cardiovascular disorders, infections, inflammatory diseases, and in healthy subjects after exercise (e.g., marathon), which indicates that the phenomenon is not exactly cancer-specific [59].

Aberrant DNA methylation is an epigenetic alteration that occurs in an organ-disease-specific manner, and therefore, it has been studied as a molecular diagnostic marker. In GC, methylated promoter regions have been widely used to identify ctDNA in both plasma and serum using methylation-specific PCR. Nowadays, frequent promoter hypermethylation and subsequent loss of protein expression have been demonstrated to be GC-related. Meta-analysis study of gastric cancer diagnosis specificity recently described that the serum hypermethylation of the APC1A and RASSF1A promoters in cfDNA was a common epigenetic event in patients with early operable GC [63]. Epigenetic alterations are thought to be an early event that possibly precedes gastric carcinogenesis, DNA hypomethylation, and CpG island hypermethylation in pre-neoplastic or early neoplastic stages and may serve as indicators or biomarkers for screening patients with an increased risk for GC. Hamakawa et al. conducted research on the possible use of ctDNA in monitoring GC disease state by targeted deep sequencing of plasma cell-free DNA by massively parallel sequencing in patients with tumor harboring [64].

In view of these research results, it is clear that changes in cfDNA/ctDNA concentrations may be a reliable biomarker in detecting the early stages of gastric cancer.

## 7. Autoantibodies against Tumor Associated Antigens

The human immune system senses the presence of cancer before manifestation of the disease. IgG autoantibodies against specific tumor associated antigens (TAAs) are found in the blood about five years before the clinical manifestation of cancer, which indicates their importance in predicting early-stage cancer. In addition, they are found in all tumor types that have been analyzed so far and they are highly antigen specific and stable [65]. Unlike the known gastric cancer biomarkers such as CA19-9, CEA, and pepsinogen, TAAs are qualitative, not quantitative, biomarkers. Assessing the autoantibody response against these autoantibodies with multiplex immunoassays is feasible and this method could make it clinically applicable [66]. The development of high-throughput proteomic techniques, e.g., various recombinant and native protein microarrays and bead-based technologies have enabled the simultaneous detection of autoantibodies against many different TAAs [67]. However, each individual biomarker of cancer-associated autoantibodies has limited diagnostic value. The frequency of antibodies against any particular antigen typically ranges from 1–15%, so autoantibodies in cancer patients are diverse [68]. The most important biomarkers for the early diagnosis of GC would be those capable of detecting neoplastic lesions in high-risk individuals. Frequently research is being carried out on the diagnostic utility of a combination of different GC-associated autoantibodies. Wu et al. showed that serum p53 protein and anti-p53 antibodies are associated with an increased risk of cancer and can be used as early serological markers in the diagnosis of malignant neoplasms [69]. However, these are not the only antibodies used as gastric cancer biomarkers. Zhang et al. detected autoantibodies against either c-myc, cyclin B1, p62, Koc, insulin-like growth factor 2 mRNA-binding protein 1 (IMP1), or survivin [70]. Xu et al. detected autoantibodies to keratin-23 (KRT23), an IQ motif containing GTPase 3 activating protein (IQGAP3), or islet-derived 3-alpha regenerating protein (REG3A) in 22.9% of gastric cancer patients [71]. Stage-specific sensitivities have been reported for antibodies against AEG-1, NY-ESO-1, p53, CTAG2, DDX53, MAGEC1, MAGEA3, and GRP78 [68]. The abundance of autoantibodies in cancer patients can be explained by overexpression, aberrant expression, mutation, or abnormal posttranslational modification of the corresponding TAAs.

Generally, in studies, the recognized indicators can distinguish gastric cancer patients from healthy with relatively high specificity (87–100%), but with discrepant sensitivity (19.3–98.9%). For example, among the most studied individual markers in gastric cancer patients, there are autoantibodies against well-known TAAs such as p53 (specificity range of 95.25–100%, and a sensitivity range of 8.1–32.1%) [68]. The biological mechanisms of the restriction of autoantibody sensitivity are still unexplained. There are several studies describing AUC: Meistere et al. reported an area under the curve (AUC) of 0.60 [72]. Zayakin et al. found that 45 autoantibodies could distinguish patients with gastric cancer from healthy individuals with an AUC of 0.79, while Zhou et al. reported that autoantibodies against seven TAAs could distinguish gastric cancer patients from healthy subjects with an AUC of 0.73 [73,74].However, these studies differ widely on various important aspects such as the method used for autoantibody detection the multiplexing level (2–45 autoantibodies), approaches used for data normalization and cut-off definition, and definition of appropriate healthy groups. Additionally, heterogeneity of TAA repertoires in cancer patients is very high, and each and single autoantibody biomarker has a generally low detection rate. Nevertheless, autoantibodies may be important in the stratification of risk group patients. One of their advantages over other biomarkers is the early detection of cancer development by the adaptive immune system [75]. Moreover, TAAs in gastric cancer patients have not yet been analyzed in the context of the IgG subclasses. Each of the IgG1-4 subclasses has different affinities for activating or inhibiting Fcγ receptors which may result in an immune response that protects the host or promotes the tumor. In addition, mucosal linings produce much more antibody type A than all other types of immunoglobulins, so TAA-specific IgA analysis may reveal new biomarkers.

## 8. Conclusions

Based on the most recent data, this review highlights the potential of newly reported molecular markers as indicators of gastric cancer and assesses their association with disease susceptibility, prognosis, diagnosis, and response to treatment. Changes in various biomarkers during cancer progression can help doctors monitor cancer status. Future improvement in treatment outcomes for GC depends on the detection of specific and sensitive biomarkers. Currently, there are no perfect tumor markers or tumor markers for gastric cancer.

High-performance biomarkers for early detection of primary outbreaks, potential metastasis and predictions of chemosensitivity enable personalized therapy. In recent years, a great deal of effort has been devoted to discovering different types of cancer-related molecules in the blood of gastric cancer patients. However, despite numerous studies on an effective indicator for predicting and detecting gastric cancer, only some showed promising results. There are high hopes for the use of liquid biopsy in the near future. CTCs, microRNAs, ctDNA, and tumor exosomes, are involved in liquid biopsies. The few biomarkers identified have extremely high sensitivity and specificity that far exceed the previously known GC serum biomarkers such as CEA, CA 19-9, and CA 72-4. Although higher levels of a biomarker can potentially predict a tumor, other factors may also account for such elevated levels. As each of these biomarkers have advantages and disadvantages, the combination of parameters may be advantageous. It seems that the best way is to determine at least two to three or more indicators simultaneously in order to increase their usefulness in diagnosing gastric cancer. It is very important to use multiple tumor markers in different types of cancer for screening, diagnosing and staging a tumor, assessing prognosis, and monitoring relapse after treatment.

New bio-liquid testing systems that combine different types of biomarkers, will be developed in the future, and will allow the collection of all information about the state of the disease, the genetic composition of the tumor, and the patient’s immune status. The alternative source of biomarker detection can be stomach juice. Many mucosal cells can be found in gastric juice, the detection of molecular markers in stomach juice is a possible noninvasive approach to screening for gastric cancer. Moreover, biomarkers are directly released by cancer cells without being excluded by the liver.

Due to large-scale research on gastric cancer biomarkers, there is a large group of these potential markers. There are many reviews that describe them, focusing on different groups of non-invasive markers [76]. This review summarizes the current knowledge, broadly describing the latest research on molecular and circulating markers that raise the greatest hopes for future use in gastric cancer diagnostics.

Unfortunately, existing clinical guidelines focusing on early diagnosis of gastric cancer do not provide consistent and prudent evidence. Establishing standard procedures and integrating new data is a great challenge. This requires the efforts of research groups to jointly develop guidelines for reporting results and standard pre-analytical and analytical procedures. We await with great interest further research on biomarkers that will improve their clinical applications in the diagnosis and treatment of gastric cancer.

## Figures and Tables

**Table 1:** Diagnostic performance of liquid biopsy in gastric cancer.

Group/Markers	Significance	Study
**Classical tumor markers**		
carcino-embryonic antigen (CEA)	Prognostic biomarker	[10,11]
carbohydrate antigen (CA 19-9)	Prognostic biomarker	[12,13]
carbohydrate antigen 72-4 (CA72-4)	Prognostic biomarker	[14]
**Circulating tumor cells**	Prognostic and monitoring biomarker Chemotherapy response	[20–24]
**Circulating free microRNAs**	Diagnosis, metastasis, monitoring response to treatment	[27–34]
**Circulating extracellular vesicles**	Diagnosis and prognostic biomarker	[43–46]
**Circulating free DNA**	Diagnosis and monitoring response to treatment	[47–53]
**Autoantibodies against tumor associated antigens**	Diagnosis and monitoring response to treatment	[58–61]

**Table 2:** microRNAs in gastric cancer [37].

Symbol	Location	Materials	Function	Biomarker
miRNA-21	(17q23.1)	Circulation	Cell proliferation, invasion	Early detection
miRNA-22	(17p13.3)	Tissue	Proliferation, migration, invasion	Early detection
miRNA-29c	(1q32.2)	Tissue	Proliferation, adhesion, invasion	Early detection
miRNA-141	12p13.31)	Tissue	migration	Early detection
miRNA-191	(3p21.31)	Tissue, circulation	Proliferation, adhesion, invasion	Early detection
miRNA-26a	(3p22.2)	Tissue	Proliferation, migration, invasion, cell cycle	Monitoring recurrences
miRNA-185	(22q11.2)	Tissue	Proliferation, metastasis	Monitoring recurrences
miRNA-196a	(17q21.32)	Tissue, circulation	Proliferation, metastasis	Monitoring recurrences
miRNA-25	(7q22.1)	Tissue, circulation	Migration, invasion	Prediction of survival
miRNA-183	(7q32.2)	Tissue	Proliferation, migration, invasion	Prediction of survival
miRNA-192	(11q13.1)	Tissue	Proliferation, migration, invasion	Prediction of survival
miRNA-17-5p	(13q31.3)	Circulating	Apoptosis, proliferation, migration, invasion	Prediction of treatment response
